# Tell me who you go with and I'll tell you who you are

**DOI:** 10.1002/hem3.36

**Published:** 2024-01-28

**Authors:** Melania Tesio

**Affiliations:** ^1^ UR LIB Lymphoma Immune‐Biology, Centre International de Recherche en Infectiologie (CIRI) Institut National de la Recherche Médicale (INSERM) Lyon France

A healthy hematopoietic system relies on proper cell fate decisions, which ensure balanced self‐renewal and differentiation programs. In this context, the dynamic regulation of the epigenetic landscape plays a pivotal role. A complex interplay of distinct chromatin factors, such as chromatin remodeling complexes and histone‐modifying complexes, regulates the expression of cell‐specific transcriptional programs driving self‐renewal, cellular differentiation, and lineage specification. Alterations in this interplay lead to an aberrant fate choice, hence contributing to the development of hematological malignancies. Whereas single‐cell technologies have contributed to understanding the role of transcriptional factors in both normal and malignant hematopoiesis, the role of chromatin factors remains less clear. In an elegant study, recently published in *Nature Genetics*, David Lara‐Astiaso and colleagues comprehensively characterized chromatin regulatory networks controlling lineage specification and differentiation during normal and malignant hematopoiesis.^1^

The authors transduced murine multipotent progenitor cells with a CRISPR library targeting 680 chromatin factors and next ex vivo exposed the cells to distinct cytokine cocktails to induce four distinct fates: self‐renewal versus differentiation, myeloid versus mega‐erythroid lineage branching, and myeloid differentiation from either multipotent progenitors or myeloid primed progenitors. To evaluate the effects of individual chromatin factors silencing on these four fate transitions, the authors next examined the single‐guide distributions in the distinct populations arising from the ex vivo culture. Using this strategy, the authors identified 143 chromatin factors affecting hematopoietic differentiation and next explored the in vivo functional role for 60 of them. Upon transplanting CRISPR‐transduced cells into recipient mice, they performed a Pertub‐seq on the cellular progeny they recovered two weeks later. This highlighted very distinct roles for chromatin factors belonging to the same family and mediating similar epigenetic activity. As an example, whereas silencing components of MLL1, a member of the COMPASS H3K4 methyltransferase, favored B‐cell fate priming over granulocytic differentiation, silencing components of MLL4, a distinct member of the same family, induced loss of hematopoietic stem cell self‐renewal, early myeloid branching, and monocyte versus granulocyte specification. Interestingly, the authors also showed that, owing to their functional diversity and multimeric nature, certain chromatin complexes switch composition during differentiation. For instance, BAF chromatin remodeling factors, which exist in three different assemblies (canonical, PBAF, and noncanonical), switch from the canonical form during early myeloid priming to the noncanonical form during terminal myeloid differentiation. By combining CHIP‐analysis, ATAC‐seq, and transcription factor footprint analysis, the authors next demonstrated that this is accompanied by a switch in the specific interactions occurring between chromatin factors and lineage‐determining transcription factors. For instance, whereas the noncanonical BAF complex interacts with a wide range of transcription factors in myeloid progenitors, its more restricted interactions with the Cebp factors are needed for terminal myeloid differentiation.

These findings exemplify some of the merits of David Lara‐Astiaso's works, which not only identified individual chromatin factors required for lineage specification but also defined a roadmap of specific transcription factor–chromatin factor interactions regulating fate decision along hematopoiesis. Importantly, these investigations were not limited to physiological conditions but extended to pathological contexts. Using a mouse model recapitulating the development of an aggressive acute myeloid leukemia (*Npm1c*/*Flt3*‐ITD), the researchers showed that leukemia cells hijack COMPASS H3K4 methyltransferases and BAF chromatin remodeling complexes to sustain their growth. Notably, however, in AML cells, both chromatin factors establish aberrant interactions with alternative transcription factors. As an example, the COMPASS MLL4 methyltransferase, which in normal cells binds to Cebpa, Nfil3, and Hlf, switches its binding to Stat5 and Runx1/2 in *Npm1c*/*Flt3*‐ITD mutant cells. Similarly, instead of binding to Pu.1 and IRF as in normal cells, canonical BAF establishes interactions with Runx1/2 (Figure 1). Highlighting a leukemia‐specific rewiring of chromatin regulatory networks, these findings raise the possibility of developing novel approaches to target aberrant chromatin factor–transcription factor associations.

**Figure 1:**
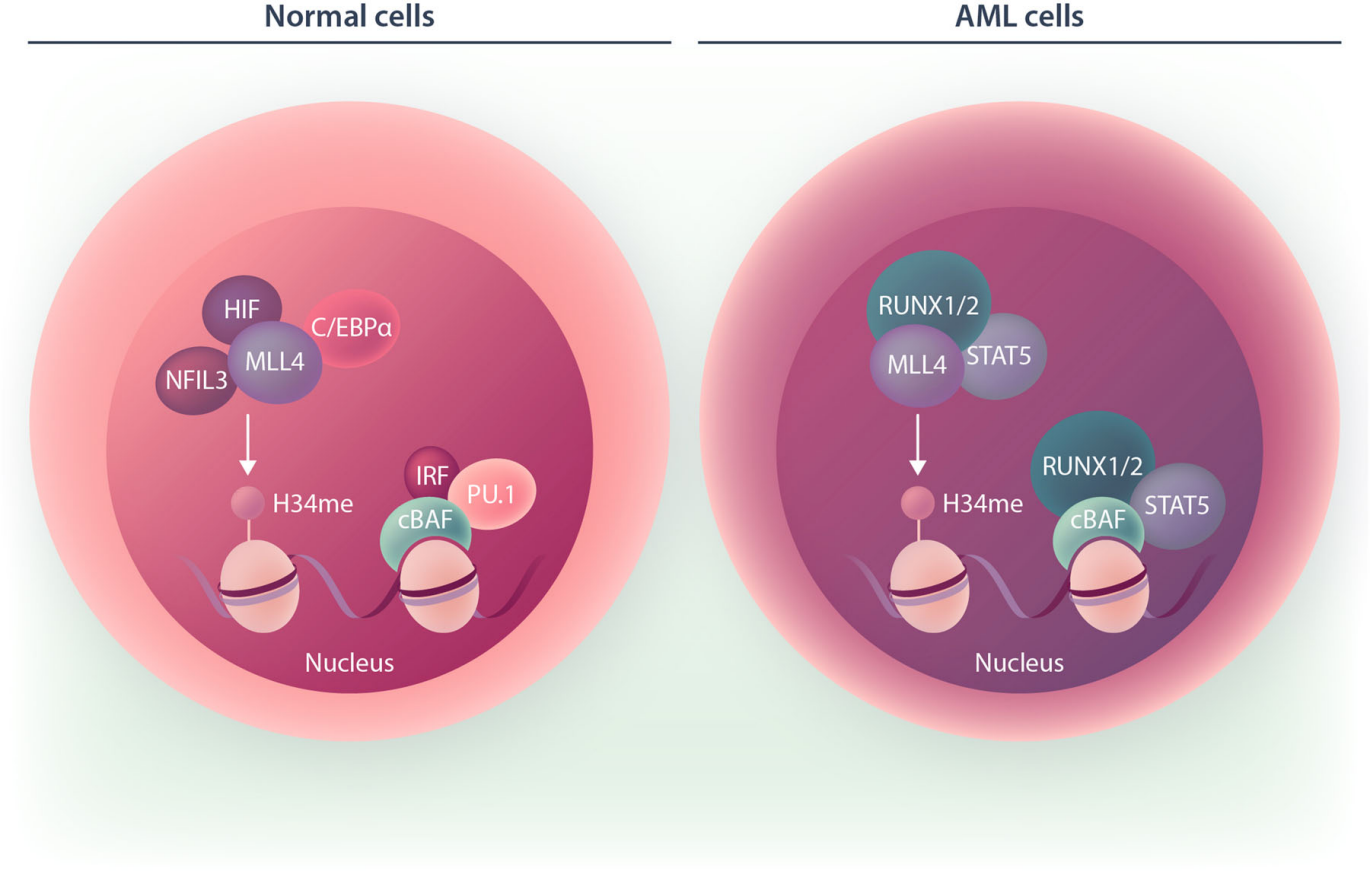
Corrupted associations between chromatin factors and transcriptional factors in AML cells evidenced by Lara‐Astiaso et al.

Providing a roadmap of these interactions during malignant and normal hematopoiesis, Lara‐Astiaso's works also open novel investigations in interdisciplinary fields, such as the metabolic control of epigenetics. Accumulating evidence has begun to demonstrate that a number of metabolic pathways produce metabolites which are either cofactors or activators of histone‐modifying enzymes.^2^ This holds true not only for “canonical” histone modifications, such as acetylation and methylation, but also for a number of novel, noncanonical modifications, such as acylations and homocysteinylation, which are emerging players in a variety of physiological contexts.^3^ Interestingly, moreover, a few metabolites have already been shown to modulate normal and transformed hematopoietic cell behaviors.^4^, ^5^ In light of this, it will be interesting to explore whether, how, and in which cellular contexts metabolic pathways impinge on the functional diversity of chromatin factors, their dynamic interactions with lineage‐determining transcriptional factors, and the leukemic rewiring of these associations. Understanding these aspects will diversify and potentiate the strategies to therapeutically interfere with corrupted chromatin regulatory networks.

## AUTHOR CONTRIBUTIONS

Melania Tesio conceived and wrote the manuscript.

## CONFLICT OF INTEREST STATEMENT

Melania Tesio is supported by a Ligue contre de cancer grant.

## FUNDING

No funding was received for this manuscript.

## Data Availability

Data sharing is not applicable to this article as no new data were created or analyzed in this study.
